# Evolution of Extra Virgin Olive Oil Quality under Different Storage Conditions

**DOI:** 10.3390/foods10081945

**Published:** 2021-08-21

**Authors:** Soraya Mousavi, Roberto Mariotti, Vitale Stanzione, Saverio Pandolfi, Valerio Mastio, Luciana Baldoni, Nicolò G. M. Cultrera

**Affiliations:** 1 Institute of Biosciences and Bioresources, National Research Council, 06128 Perugia, Italy; soraya.mousavi@ibbr.cnr.it (S.M.); saverio.pandolfi@ibbr.cnr.it (S.P.); luciana.baldoni@ibbr.cnr.it (L.B.); niccolo.cultrera@ibbr.cnr.it (N.G.M.C.); 2 Institute for Agricultural and Forest Systems of the Mediterranean, National Research Council, 06128 Perugia, Italy; vitale.stanzione@cnr.it; 3 Estación Experimental Agropecuaria San Juan, Instituto Nacional de Tecnología Agropecuaria (INTA), Consejo Nacional de Investigaciones Científicas y Técnicas (CONICET), Ing. Marcos Zalazar (Calle 11) y Vidart. Villa Aberastain, Pocito, San Juan 5427, Argentina; mastio.valerio@inta.gob.ar

**Keywords:** extra virgin olive oil, storage, oxidation, phenols, sterols, tocopherols, temperature, argon

## Abstract

The extent and conditions of storage may affect the stability and quality of extra virgin olive oil (EVOO). This study aimed at evaluating the effects of different storage conditions (ambient, 4 °C and −18 °C temperatures, and argon headspace) on three EVOOs (low, medium, and high phenols) over 18 and 36 months, analyzing the main metabolites at six time points. The results showed that low temperatures are able to maintain all three EVOOs within the legal limits established by the current EU regulations for most compounds up to 36 months. Oleocanthal, squalene, and total phenols were affected by storage temperatures more than other compounds and degradation of squalene and α-tocopherol was inhibited only by low temperatures. The best temperature for 3-year conservation was 4 °C, but −18 °C represented the optimum temperature to preserve the organoleptic properties. The present study provided new insights that should guide EVOO manufacturers and traders to apply the most efficient storage methods to maintain the characteristics of the freshly extracted oils for a long conservation time.

## 1. Introduction

Extra virgin olive oil (EVOO) is one of the most valuable vegetable oils and its consumption has now expanded worldwide owing to its unique flavor and richness in bioactive compounds, such as phenols, tocopherols, squalene, and sterols [1,2,3].

Various factors may act on the characteristics and chemical composition of EVOOs, including varieties, orchard management, harvesting time, oil extraction, and storage conditions [4,5]. In particular, storing can greatly affect the oxidation stability of EVOO, one of the primary causes of its degradation, which can progress in the dark (autoxidation), and can be accelerated in the presence of light (photo-oxidation) and enzymes (enzymatic oxidation) [6]. The high oleic acid content and the natural antioxidants present in EVOOs, including phenolic compounds, tocopherols (mainly α-tocopherol), squalene, chlorophyll, carotenoids, and vitamins, are tied to oil stability by providing an effective defense against free radicals by different mechanisms [7,8,9,10,11]. Minor polar phenolic compounds are constituted by simple phenols, such as tyrosol and hydroxytyrosol, and by their combination with other moieties to form oleuropein and ligstroside and their derivatives, cinnamic acids, as well as lignans and flavonoids [12]. It was demonstrated that the degradation rate of phenolic compounds during oil aging is strongly related to their initial concentration [13]. Some of the simple phenols, such as tyrosol and hydroxytyrosol, increase over time, likely owing to hydrolytic processes of secoiridoid derivatives representing their linked forms [14]. The percentage of hydrolysis typically increases in aged EVOO; therefore, the EVOO quality should be correlated to lower and not to higher values of tyrosol and hydroxytyrosol [15]. Tyrosol and hydroxytyrosol increase has been observed in EVOOs stored for three months at room temperature [16], and a high decrease in secoiridoids (close to 50%) was observed in EVOOs with low initial minor polar compounds after 18 months of conservation at room temperature, while in oils with a high initial amount, the decrease was close to 20% [17]. A decrease in phenol content was reported in EVOOs stored at 1 °C for 12 months, although lignans were more stable in the same conditions [15].

The effects of the storage conditions on acidity, peroxide value, and fatty acid profile as criteria of the EVOO quality have been studied in different types of containers and at different temperatures, from 5 to 25 °C [13,15,16,18]. High-quality EVOOs stored at 12−15 °C showed a reduced lipid oxidation [16,19] and peroxide value and acidity, and delta-K did not change in EVOOs conserved at 0–8 °C for 24 months [13]. The autoxidation stability of several EVOOs in 21 months of storage in dark and at room temperature showed that the antioxidant concentration directly affected the extinction coefficient K_232_, correlated to product oxidation [20]. In a recent study, it was reported that K_232_ could be used as an effective and cheap measure to monitor the quality evolution during storage [18]. Fadda et al. [21] found only 13% α-tocopherol degradation in EVOOs over 18 months of storage in the dark, while Okogeri and Tasioula-Margari [22] reported up to 79% loss of α-tocopherol after 4 months of storage under light, and nearly complete loss over 12–24 months of storage [23].

Squalene is a rather stable molecule in the absence of oxygen and has a weak antioxidant activity, owing to competitive oxidation with the lipid substrate [24]. In a study on degradation of squalene during EVOO storage, oil samples were stored in dark and colorless bottles, filled completely or halfway, in order to simulate the domestic storage conditions. The content of squalene decreased significantly only after 6 months in half-empty bottles, and diffused lighting did not play a significant role in squalene degradation after 12 months of storage in filled colorless bottles under lighting [25]. Nevertheless, the contribution of squalene to olive oil stability under light exposure or in the dark, scarcely investigated up to now, would deserve further attention to obtain conclusive results [8].

Phytosterol isomers decrease during EVOO storage and sterol oxidation increase through their reaction with light, high temperature, and oxygen, and this increment depends on their unsaturation level [26]. A high concentration of chlorophylls increases the stability of EVOO exposed to light and oil photo-oxidation in the presence of chlorophylls, leading to the formation of highly unstable and reactive singlet oxygen that tends to react with the unsaturated fatty acids, leading to the formation of hydroperoxides. Carotenoids are effective inhibitors of photo-oxidation by quenching singlet oxygen and triplet excited states of photosensitizers [7].

Most EVOO producers and researchers consider 12−18 months as the maximum commercial storage period from bottling to consumption and current guides on best practices delivered by the International Olive Council (COI/BPS/Doc. No 1—Best practice guidelines for the storage of olive oils and olive-pomace oils for human consumption, guide No 1/2018) suggest the commercialization within 24 months, but 18 months is still commonly reported as the “best before” date since bottling.

While extensive information is available on EVOO storage at high and ambient temperatures and light exposure, few works have been performed on the effects of low and freezing temperatures [6,15,16] in which the effects of low temperatures were studied on few oil compounds, mostly just phenols, and the extent of the experiment was limited to 18 months. The present study aims to evaluate how different storage conditions (ambient temperature, with and without argon, and cold treatments at +4 °C and −18 °C) on three EVOOs with different initial phenol content may preserve chemical and organoleptic characteristics over a long and very long time of conservation (18 and 36 months).

## 2. Materials and Methods

### 2.1. Materials and Storage Conditions

EVOOs with low phenols (LP, up to 200 mg kg**^−^**^1^), medium phenols (MP, up to 400 mg kg**^−^**^1^), and high phenols (HP, more than 400 mg kg**^−^**^1^) were provided by a quality-assured industrial oil mill. After filtration, oils were stored in 250 mL amber glass bottles, closed with hermetic caps and maintained in the dark for all treatments and along the entire storage duration.

Four different treatments were settled for storing the bottles (12 bottles for each oil in each storage condition): (1) ambient temperature (AT); (2) ambient temperature + argon saturated headspace (AT + Ar) (excepting for HP oil); (3) refrigerated at 4 ± 1 °C; and (4) freezed at −18 ± 1 °C. In treatments 1, 3, and 4, the headspace was occupied by unmodified atmosphere. Two different storage durations were considered: 18 months (long-term storage, T18) and 36 months (2× long-term storage, T36) (Figure S1).

The effects of the storage conditions on oil quality were evaluated at the following time points: at the beginning of treatment (T0) and after 18 months storage (T18). To analyze the effects of 18 months of storage on the posterior oil stability, oil samples were evaluated at 72 h (T18-72 h), one month (T18-1 m), and eight months (T18-8 m) after treatment completion and bottle opening. After each time point, the oil bottles were closed and placed in the dark condition at ambient temperature for the next time point. Bottles were closed as in everyday use; no special procedure was done to remove oxygen. Through these analyses, we tried to simulate the gradual usage of oil and its progressive oxidation that usually happen after a long storage period and its usage by the consumers.

To study the effects of 2× long-term storage (T36), EVOO bottles of each treatment were kept closed for the entire storage period (Figure S1).

Six bottles for each oil, storage condition, and time point were used, three for chemical analysis and three for organoleptic evaluation. Samples stored at 4 °C and −18 °C were taken out from refrigerators and maintained at room temperature for 6 h, in order to thaw oil at liquid status.

### 2.2. Quality and Oxidation Indices

Acidity was measured and expressed as a percentage of free oleic acid. Peroxide value (PV) was stated as milliequivalents of active oxygen per kilogram of oil (meq O_2_ kg**^−^**^1^). K_232_ and K_270_ extinction coefficients (UV absorbance) were calculated from absorption at the exact λ wavelengths in nm, following the analytical methods described in the European Commission Regulation EEC 2568/91 (Regulation, 1991) and later amendments (the latest being EU 1348/2013).

### 2.3. Analysis of Total Phenolic Compounds

The content of total phenols was determined by the Folin–Ciocalteu (FC) method, according to the analytical protocol described by Singleton et al. [27]. The method was adapted for oils as follows: 5 g of oil was extracted with 5 mL of methanol/water (80:20 *v*/*v*) by 30 min shaking and 5 min centrifugation (1700× *g*). A 1 mL extract was added to 0.25 mL of FC reagent and 1.5 mL of Na_2_CO_3_ (20% *w/v*), in a 10 mL volumetric flask, reaching the final volume with purified water. Each sample was stored for 90 min at a controlled temperature of 25 °C in dark conditions, and the spectrophotometric analysis was performed at λ = 725 nm. The results, expressed in mg kg**^−^**^1^ of gallic acid (GA), were obtained through a calibration curve ranging from 1 to 15 μg mL^−1^ (R^2^ = 0.9985).

### 2.4. Extraction of the Phenolic Fraction

Following the method described by the International Olive Council (Method COI/T.20/Doc. No 29, 2009), 5 g of olive oil was added to 5 mL methanol/water (80/20 *v*/*v*) and 100 μL of syringic acid as an internal standard (IS) was added to each sample. Samples were shaken for 1 min to homogenize the mixture and then centrifuged at 2200× *g* for 25 min at 4 °C. Finally, the supernatant was injected into the HPLC-DAD system. An external standard method was used in quantifying the phenolic compounds using their related calibration curves and the contribution of the internal standard.

### 2.5. Analysis of Phenolic Compounds through Reverse-Phase HPLC

The HPLC analyses of the phenolic extracts were conducted according to Selvaggini et al. [28], with a reversed-phase column using a HPLC-DAD, Varian ProStar-Diode Array Detector 330. The machinery was composed of a vacuum degasser, a quaternary pump, an autosampler, a thermostatic column compartment, and a DAD, using a 250 × 4.6 mm column 5 µm Kinetex EVO C18 100A (Phenomenex, Torrance, CA, USA). Eluent “A” was made with water and phosphoric acid 0.2% (Carlo Erba, Milano, Italy) and eluent “B” was methanol/acetonitrile (Carlo Erba) 50:50 (*v*/*v*). The elution gradient started from 4% eluent B and reached 100% B after 55 min for 15 min at a flow rate of 1.2 mL min**^−^**^1^. Phenolic compounds were quantified at three wavelengths: 280, 310, and 360 nm using an authentic external standard. Secoiridoid derivatives, oleacein (3,4-DHPEA-EDA), and oleocanthal (p-HPEA-EDA) with 95% purity were provided by Prof. P. Magiatis (University of Athens, Greece). All other identified phenols were purchased from Sigma-Aldrich (St. Louis, MO, USA).

### 2.6. Analysis of Tocopherols

Tocopherol composition was determined by modifying the HPLC procedure described in [29]; 0.15 g of olive oil was dissolved in 5 mL hexane and homogenized by stirring. Samples were analyzed using HPLC-DAD 330 and the same column as for the phenolic compounds. The calibration curve was obtained by injecting standard solutions of (±)-α-Tocopherol-synthetic, ≥96% (Sigma-Aldrich, St. Louis, MO, USA) at different concentrations. The HPLC analysis was performed using a mobile phase composed by wluent “A” water with phosphoric acid 0.2% (Carlo Erba) and eluent “B” was methanol (Carlo Erba), at a ratio A/B 10:90. The flow rate was 1.2 mL/min, the injection volume was 30 µL, and the time of analysis was set for 20 min. Detection and quantification were performed at 290 nm.

### 2.7. Sterol and Squalene Analyses

Around 200 mg of each sampled oil was placed in a 10 mL propylene tube. Then, 200 μL of an internal standard solution (5α-cholestan-3β-ol, Sigma-Aldrich) in hexane was added. The analysis of the unsaponifiable fraction was performed by GC, without a preliminary thin-layer chromatography fractionation. This approach was used for the analysis of sterols and squalene in olive fruit and olive oil by many authors [30,31]. Alkaline hydrolysis was performed by adding 2 mL of KOH 2%, then tubes were soaked in a water bath at 80 °C for 15 min, and the unsaponifiable fraction was extracted by vortexing with 1 mL hexane and 1.5 mL NaCl 1%. The upper hexane layer was transferred to 2 mL glass vials. Samples were conserved at −20 °C until analysis, within 24 h from preparation.

Analyses were performed on a GC-FID, using a ZB-5HT Inferno capillary column (15 m × 0.32 mm × 0.10 μm film thickness, Phenomenex, Torrance, CA, USA). The carrier gas was helium (column flow 1.5 mL/min), and the split ratio was 1:100. The oven temperature was programmed as follows: 0.5 min at 150 °C, from 150 °C to 240 °C at 8 °C/min and from 240 °C to 370 °C at 25 °C/min held for 5 min, at 370 °C, followed by 320 °C for the injector and 350 °C for the detector (FID). The quantification was performed by external standards of squalene and sterols purchased from Sigma-Aldrich (St. Louis, MO, USA).

### 2.8. Analysis of Chlorophylls and Carotenoids

Chlorophylls and carotenoids were determined at 670 nm and 470 nm, respectively, following Minguez-Mosquera et al. [32] protocol. The oil samples were dissolved in cyclohexane (1.5:5 *w/v*) and absorbance was measured using a Perkin Helmer Lambda 10 UV–vis spectrophotometer.

chlorophylls = (A670 × 106)/(613 × 100 × d)(1)

carotenoids = (A470 × 106)/(2000 × 100 × d)(2)

where A is the absorbance and d is the path length of the cell (1 cm).

### 2.9. Fatty acid Methyl Ester (FAME) Analysis

Approximately 150 μL of oil in 2 mL of hexane was trans-methylated with 200 μL of a cold solution of KOH in methanol (2 M), according to the European Standard NF EN ISO 12966-2 [33]. Fatty acid methyl esters (FAMEs) were analyzed in accordance with the European Standard NF EN ISO 5508. Analyses were performed on a Varian Gas Chromatograph CP3800 equipped with the flame ionization detector (GC-FID) (T = 320 °C), using a capillary column (60 m × 0.25 mm i.d., 0.25 μm film thickness) coated with polyethylene glycol (Zebron, ZB-WAX, Phenomenex, Torrance, CA, USA). The carrier gas was helium (column flow 1.5 mL**/**min) and the split ratio was 1:100. The oven temperature was programmed as follows: 2 min at 140 °C, increased from 140 °C to 240 °C at 4 °C/min, held for 15 min, then 42 min at 240 °C. FAMEs were identified by comparing the retention times with the standard solution of Supelco 37 Component FAME Mix (Sigma-Aldrich, St. Louis, MO, USA).

### 2.10. Organoleptic Evaluation

The organoleptic profile of the oil samples was evaluated at all time points (T0, T18, T18-72 h, T18-1 m, T18-8 m, and T36) by the panel taste of the Slow Food organization, following the IOC method for the organoleptic assessment of virgin olive oil (COI/T.20/Doc. No 15/Rev. 10 2018) with some modification. The most important differences concerned the number of tasters, which was five plus a leader, and the number of analyzed oil per section, which was five out of four, as reported in the IOC method, and all the other recommendation were applied. The scale used for each olfactory and gustatory sensation was from 0 to 10 for each perception. The presentation of each sample to the panelist was blind. The tasting session begins with a calibration between the members, with the panel leader, who provided different oils and evaluated the response of each member. After the calibration, the panel leader, in a separate room, numbered every replica of oil and then offered to the panel members one glass with the same code at a time with a maximum of five oils for each section. Moreover, the panel leader checked the results, performing the average of them, and controlled if reported results were out of range. If this last case occurred, the panel leader started a new calibration test and the sample was numbered and tasted again. Olfactory and gustatory sensations were evaluated considering positive attributes, i.e., fruitiness, bitterness, pungency and persistence, and negative traits, as the presence of defects (i.e., fusty/muddy sediment, musty/humid/earthy, winey/vinegary/acid/sour, rancid, and others). The panel leader compiled the notes given by each taster and the statistical evaluation was carried out by the median of each parameter. This test provided sequential information about the sensory characteristics of the samples and allowed identifying changes in the organoleptic profile of oils under different treatments and time points.

### 2.11. Statistical Analysis

Data were analyzed by DAASTAT [34] using one-way ANOVA (*p* < 0.01 = ** and *p* < 0.05 = *, *n* = 3), separately for the three oils and among different time points. Tukey test was used to compare mean values. A principal component analysis (PCA) was applied for a total of 22 chemical variables using the percentage of reduction in content of chemical parameters after long term storage (from T18 to T18-8 m) and among T18 and T36 conditions, using PAST software version 4.03 [35]. A boxplot was made by the same software to show the variability of each chemical parameters for the studied samples and treatments.

## 3. Results and Discussion

### 3.1. Free Acidity, Oxidation Indices, and Total Phenolic Content

**Figure 1:**
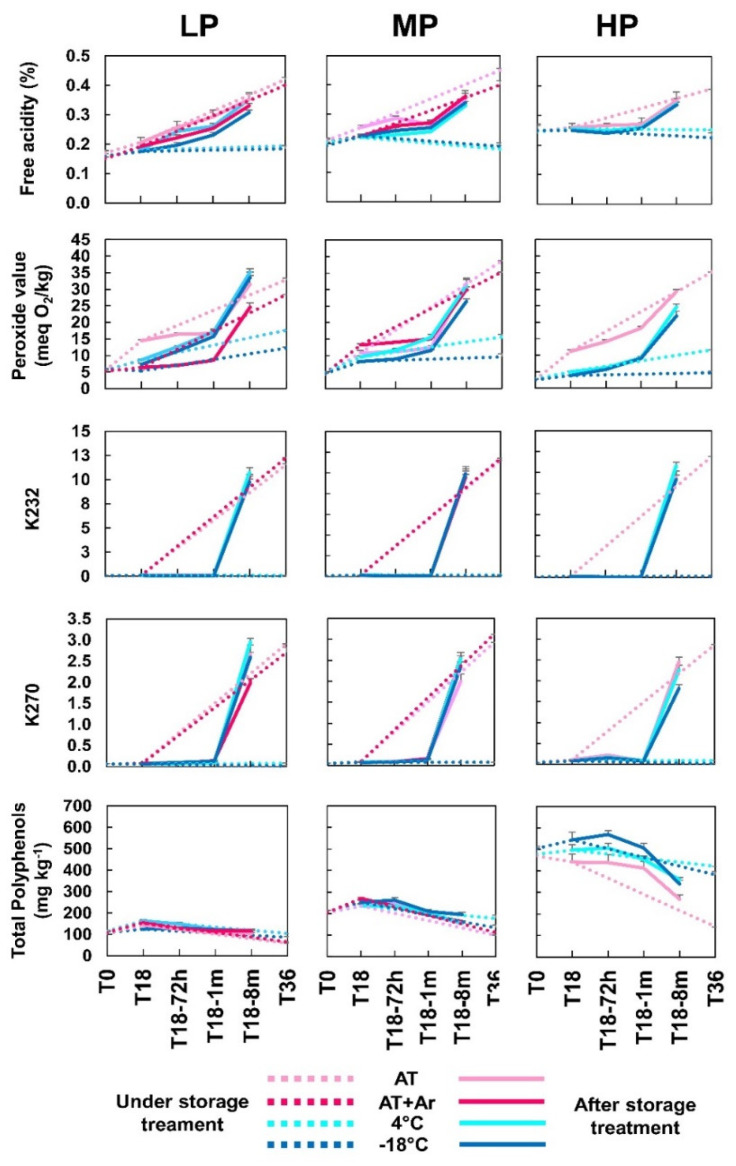
Free acidity (percentage of oleic acid), peroxide value (meq O_2_/kg), K_232_ and K_270_ extinction coefficients, and total polyphenols (mg kg^−1^ of oil) of low, medium, and high phenolic (LP, MP, and HP, respectively) EVOOs under different storage conditions and time points. Dot lines: T0, T18, and T36 (under storage treatment); solid lines: T18-72 h, T18-1 m, and T18-8 m (after storage treatment). Bars represent means ± SD of three replicates.

Analyses were performed to monitor the evolution of EVOOs’ composition stored under four different treatments (temperatures/atmospheres) along time, measuring the oxidative stability and total phenols (Figure 1).

From an initial level of free acidity (FA) ranging between 0.15 and 0.25 for the three oils, a slight increase was detected at T18 in LP and MP oils. The same trend was observed after one month of bottle opening (T18-1 m), and at T18-8 m, values rose rapidly above the maximum values of EVOO quality parameter (EU Reg. 2015/1830) (Figure 1, Table S1). It was evident that storage at low temperatures has guaranteed the maintenance of low levels of FA in all oils, but after opening bottles, the FA level increases as in the oils stored at room temperature.

Peroxide values rose during storage at AT in all three EVOOs, but argon headspace allowed to maintain very low levels, similar to frozen oils, during the first month after treatment completion, and then increased as in bottles without argon. Thus, cold temperatures maintained PV at low levels, with −18 °C being more effective than 4 °C. Very interestingly, also after 36 months of storage (T36), low temperatures allowed to keep PV levels lower than 20 meq O_2_ kg^−1^ and, in the case of HP oil, the level remained confined to around 11.6 meq O_2_ kg^−1^ for cooled oil and 4.7 meq O_2_ kg^−1^ for frozen oil. In general, only storage at low (4 °C) or very low (−18 °C) temperatures avoided the increase of PV, but at T18-8 m, all oil samples exceeded the permitted limits of PV (Figure 1, Table S1). These results are in accordance with the observations of Mulinacci et al. [16], who reported that PV remained below 9 meq O_2_ kg^− 1^ in frozen oils, while in samples stored at room temperature after nine months, its vaue rose to 21.2 meq O_2_ kg^−1^.

At T0, all EVOOs showed almost undetectable values of oxidation indices K_232_ and K_270_. These levels remained very low over 18 months of storage under any condition, but later, at T18-1 m, a sharp increase was detected in all cases, until they reached values around 10 for K_232_ and from 2 to 3 for K_270_ at T18-8 m, exceeding the legal limits for both parameters (Table S2). Moreover, oils stored at AT or AT + Ar deteriorated progressively; meanwhile, storage at 4 °C or −18 °C was very effective in preserving the oils, which showed very low levels of both parameters (Figure 1).

Similar results were obtained in previous studies, showing an increase of FA and PV along with storage at room temperature [9,18,36], but on the contrary, it has been observed that most of stability parameters, notably K_270_, are directly affected by ambient temperatures [37,38].

The use of argon in the bottle headspace under AT condition was effective in maintaining low FA and PV, as previously shown [39], whereas no effects were observed on other stability parameters, such as K_232_ and K_270_.

Oil samples maintained under AT and AT + Ar storage conditions for 36 months were excluded from further analyses because their quality and oxidation indices exceeded the legal limits of EVOOs (Tables S1 and S2).

The EVOOs used for the analyses were distinguished based on their content in total phenols before storage: ~450 mg kg^−1^ for HP oil, ~200 mg kg^−1^ for MP oil, and ~100 mg kg^−1^ for LP oil. After 18 months of storage, total phenols showed a slight, but not significant increase for all treatments and oils. These values did not change during the 8 months after treatment completion for LP and MP oils, whereas in HP oil, phenols significantly decreased to 270–310 mg kg^−1^ for all treatments, even though their total content remained higher than that of the other two oils (Figure 1, Table S1).

After 36 months storage, LP and MP oils stored at ambient temperature, either with or without controlled atmosphere, lost about half of their phenolic content, whereas the HP oil lost more than 70%, whereas at 4 °C and, to a lesser extent, at −18 °C, phenols did not undergo any significant changes in all EVOOs. Thus, phenol content can be well preserved at low temperatures such as 4 °C and −18 °C even after T36, regardless of their initial content (Figure 1, Table S1). Similar results have already been obtained for storage periods up to 18 months [15], but, to the best of our knowledge, we show the first evidence that oil samples stored for 3 years can retain a good phenolic profile if kept at low temperatures, and that there is no need to use lower temperatures below 4 °C.

### 3.2. Phenolic Compounds

**Figure 2:**
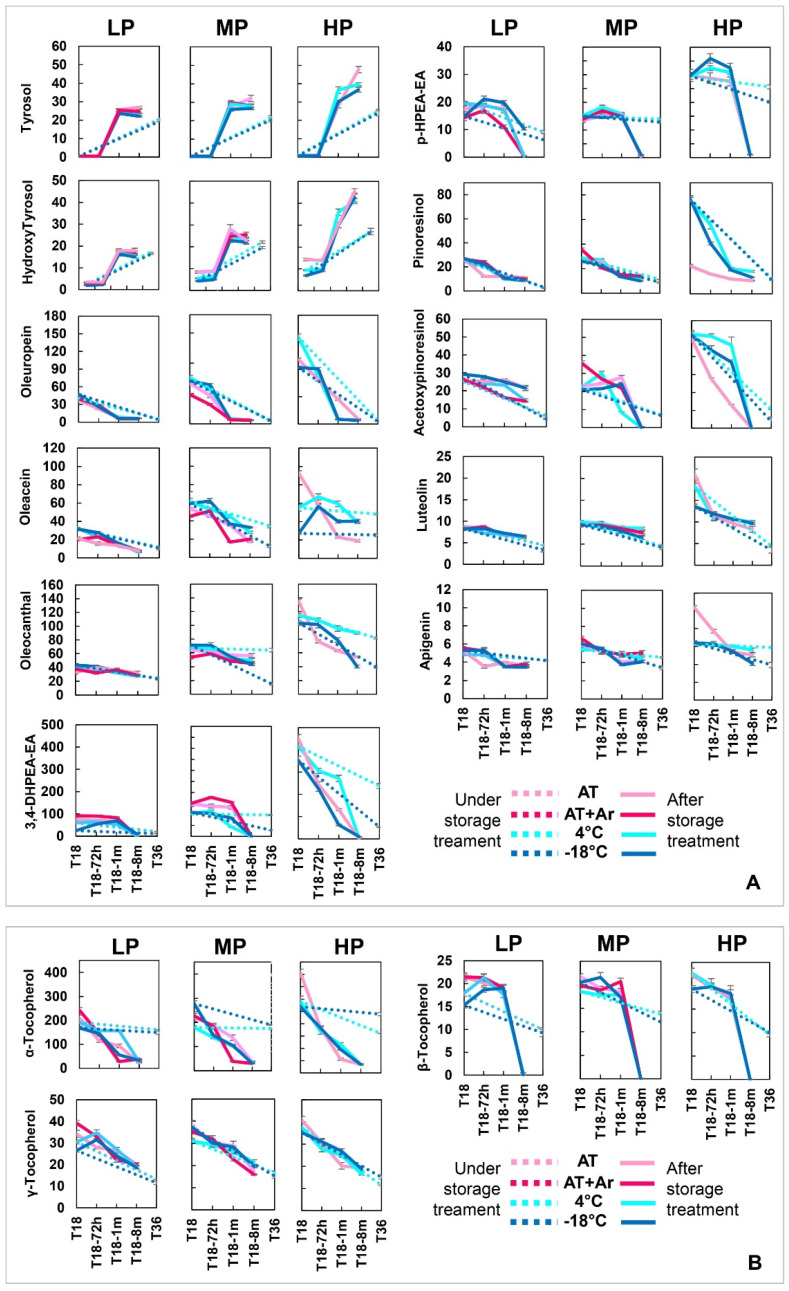
Evolution of phenolic compounds (**A**) and tocopherols (**B**) in LP, MP, and HP EVOOs under different storage conditions and time points. Dot lines: T18 and T36 (under storage treatment); solid lines: T18-72 h, T18-1 m, and T18-8 m (after storage treatment). All values are expressed in mg kg^−1^. Bars represent means ± SD of three replicates (*n* = 3).

In order to evaluate if and how storage conditions can modify the content of main phenolic compounds and the evolution of their derivatives, detailed analyses were performed on the EVOOs for all storage treatments (Figure 2, Table S3).

Simple phenols, such as tyrosol and hydroxytyrosol, remained very low at 18 months of conservation under all storage treatments, and significantly increased after storage completion and bottle opening. The same increase was observed in closed bottles conserved for 36 months (Figure 2, Table S3). This trend is probably due to the hydrolytic and oxidative degradation processes of main secoiridois (oleuropein, oleacin, oleocanthal, 3,4-DHPEA-EA, and p-HPEA-EA) [40,41], which in fact decreased significantly under all conditions. It is widely recognized that tyrosol and hydroxytyrosol typically increase in aged EVOOs [6,9,15,16,42] and, considering that hydroxytyrosol represents an important bioactive compound for human health, with a strong antioxidant activity [43,44], a low concentration of this molecule, when balanced by a high concentration of complex phenols, may represent a good compromise for EVOOs from a quality standpoint. The relationship between complex and simple phenolic compounds also depends on the initial content and processing conditions [5,45,46]. In fact, different contents of hydroxytyrosol were observed at T18, depending on the oil and storage conditions, showing the highest values in HP oil stored in AT. Eight months after bottles’ opening, the highest tyrosol and hydroxytyrosol content was measured in HP under AT condition, the same oil and condition in which the PV and FA were higher than others (Figure 2, Table S1).

The two main families of complex phenolic compounds of EVOOs are 3,4-DHPEA-EDA (oleacein) and 3,4-DHPEA-EA (oleuropein aglycon), representing the dialdehydic and aldehydic forms of elenolic acid linked to hydroxytyrosol, and p-HPEA-EDA (oleocanthal) and p-HPEA-EA (ligstroside aglycon), the dialdehydic and aldehydic forms of elenolic acid linked to tyrosol, respectively. In general, phenol reduction was more evident within secoiridoid derivatives, indicating a more active participation in the oxidative processes. On average, the best 18 months storage condition for secoiridoids’ conservation was 4 °C in all three EVOOs, especially for oleuropein, while at −18 °C, the two phenols oleacein and 3,4-DHPEA-EA showed a severe decay (25% and 48%, respectively). Oleuropein showed a significant decrease in all EVOOs and conditions, while oleocanthal was the most stable secoiridoid. The aldehydic forms of secoiridoids, such as oleuropein aglycone (3,4-DHPEA-EA) and ligstroside aglycone (p-HPEA-EA), almost disappeared eight months after treatment completion, independently from storage conditions. After 36 months, these two forms did not show a drastic reduction, but the reduction was significant under −18 °C treatment (Figure 2, Table S3); no significant differences were observed for the analyzed secoiridoids between AT and AT + Ar. The highest decrease was observed in LP oil at −18 °C followed by AT for 3,4-DHPEA-EA, p-HPEA-EA, p-HPEA-EDA, and oleacein.

Freezing temperatures led to changes in the oil physical state that passed from liquid into a solid state with a porous structure [47]. The presence of air and water through the whole volume of frozen oil could lead to a greater oxidative deterioration, and thus to a high loss of phenols [16,23]. It is important to notice that almost all secoiridoids, especially in MP and HP oils, had an increase after 72 h from 18 months storage, and the rise was higher for storage at low temperatures. It can be hypothesized that the equilibrium among secoiridoids within the oil is temperature-dependent, and the relative amounts of the isobaric forms are different in frozen and unfrozen oils. This different equilibrium can affect the measured absorbance values at 280 nm, the wavelength widely used in all HPLC/DAD methods for their determination [16]. Moreover, it is well known that the presence of a high amount of oxygen can accelerate the oxidation of phenolic compounds in the EVOOs and, consequently, it seems impossible to obtain a real increase in secoiridoids under these experimental conditions. On the other hand, the statistically significant differences between values immediately after 18 months storage stop (T18) and after 72 h (T18-72 h), observed for the peroxide values in both frozen and unfrozen oils, as expected, confirmed an increase of the primary products of autoxidation, being well known that a high amount of oxygen in the bottle headspace accelerates this process [16].

Lignan phenols (pinoresinol and acetoxypinoresinol) decreased in all EVOOs under any storage condition, as reported in other studies performed at room temperature [5,18,48]. MP and HP oils 18 months after treatment completion had a continuous loss of acetoxypinoresinol, at a maximum after eight months (93%), while storage at 4 °C was the best to conserve pinoresinol, especially when compared with the AT condition. The highest decrease of lignans was observed in HP oil under any storage condition (Figure 2).

Flavonoids (apigenin and luteolin) showed a slight decrease in LP and MP oils from T18 to T18-8 m, whereas both had a sharp decrease in HP oil. In oils stored for 36 months, luteolin decreased more at low temperatures than apigenin (Figure 2). Other authors reported the reduction of flavonoids after 12 months of storage at room temperature [5].

In order to evaluate the percentage of reduction in the content of all eleven phenolic compounds from T18 to T18-8 m and from T18 to T36, a PCA was performed on all phenolic compounds, oil samples, and storage conditions (Figure S2). The three oil samples stored at 4 °C, especially HP and MP oils, showed the lowest phenol oxidation. These results are in accordance with recent studies reporting that EVOOs with high oleacein and oleocanthal content have undergone a greater decrease in phenolic content than oils rich in other phenols [9,49]. Furthermore, after 18 months of storage at 4 °C, great stability of phenols was observed in all three EVOOs. Indeed, storage at AT + Ar in LP and MP oils was similar to that at 4 °C, in terms of stability of total phenols. It is interesting to notice that, even for 36 months of storage, HP and MP oils had the lowest phenol oxidation under the 4 °C storage condition. These results have definitely stated how storage at low temperature, when joined with low oxygen and low light availability, may allow for good preservation of the most important phenolic compounds, regardless of their initial content.

### 3.3. Tocopherols

Tocopherols were well preserved in all EVOOs after 18 months of storage at AT + Ar and at AT, but they declined after bottle opening. Among them, α-tocopherol decreased harshly at T18-8 m (89–91% in all oils), whereas β-tocopherol, albeit present in small quantities, had a significant decrease in all samples and storage conditions, and at T18-8, m dropped to zero, while the reduction of γ-tocopherol was lower than that of the other ones, with a very similar pattern for all EVOOs. In contrast, for 36 months of storage, α-tocopherol remained approximately the same, whereas β and γ-tocopherols decreased slightly (Figure 2, Table S3). Indeed, tocopherol loss was higher than that observed for polar phenols, in agreement with the results of Fregapane and Salvador [12]. The importance of tocopherols as antioxidants, by the induction period of oxidation, was revealed by a 9-month study conducted by Samaniego-Sanchez et al. [19], showing that tocopherols fell around 70–90% in all oils after storage, with higher losses in oils stored at 20 °C over those stored at 4 °C. In another study, an almost total loss of α-tocopherol in oils stored for 12 months in dark glass bottles at ambient temperature was reported [50], attributing the substantial loss of tocopherols during the storage to their function as hydrogen donors or oxygen quenchers, to stop the chain mechanism of auto- and photo-oxidation, respectively.

The good stability of α-tocopherol at low temperatures allows to hypothesize that freezing could decrease the oxidation process of tocopherols [16]. In general, the reduction in α-tocopherol suggested a protective effect of hydrophilic phenols probably based on their ability to reduce its oxidized forms [51]. In fact, the α-tocopherol value was lower than oleuropein and the aldehydic forms of elenolic acid linked to tyrosol and hydroxytyrosol (Figure 2).

### 3.4. Squalene and Sterols

**Figure 3:**
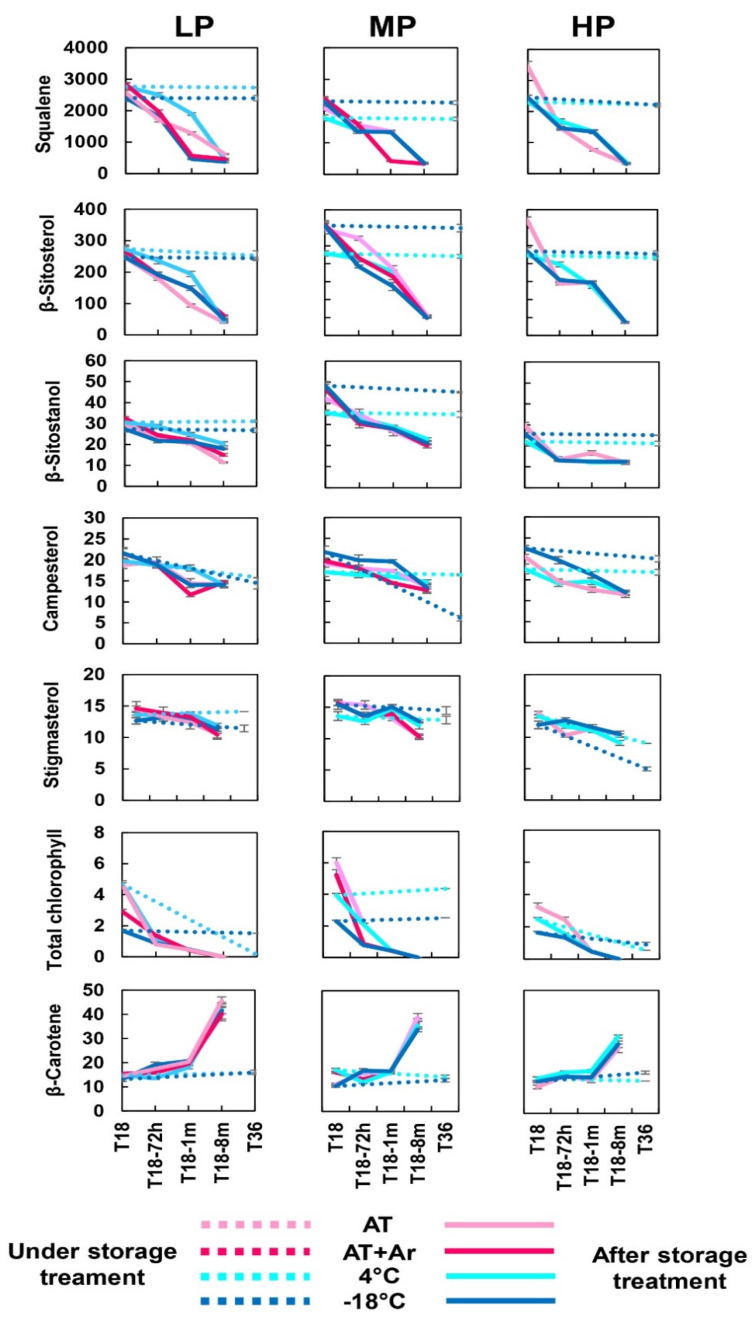
Changes of squalene, sterols, chlorophyll, and β-carotene in LP, MP, and HP EVOOs under different storage conditions and time points. Dot lines: T18 and T36 (under storage treatment); solid lines: T18-72 h, T18-1 m, and T18-8 m (after storage treatment). All values are expressed in mg kg^−1^. Bars represent means ± SD of three replicates.

After 18 months of storage, squalene was better preserved in HP oil under AT storage. After stopping the storage and bottles’ opening, a sharp decrease in squalene was reported in all EVOOs for all treatments (Figure 3, Table S4). The decrease was from a minimum of 76% in LP oil to a maximum of 91% for HP oil at ambient temperature. The very fast decline occurred 72 h after treatment completion, and then the squalene content remained approximately constant for one month, followed by a second decline to the minimum levels after 8 months. Naziri et al. [24] and Rastrelli et al. [25] reported squalene degradation along time under room temperature. On the contrary, storage at 4 °C and −18 °C preserved the level of squalene over 36 months.

Squalene loss during oil storage was similar to that of α-tocopherol and, to a lower extent, to that of phenolic compounds. Squalene and tocopherols are considered important contributors to oil oxidative stability in the dark (autoxidation), under light exposure (photo-oxidation), and upon heating [7,8].

After 18 months of storage, sterol content was similar for all three oils under any storage condition (Figure 3), but after treatment completion, β-sitosterol had the highest decrease, up to 89%, while stigmasterol had the minimum loss among sterols under −18 °C storage condition (Figure 3, Table S4).

All analyzed sterols were quite well preserved after 36 months of storage treatments. Similar results were reported by Woranik and Rekas [52], showing that the loss of total sterols in oils kept closed over the entire storage period was negligible. Our results confirmed that cold temperatures are particularly effective for preserving the sterols present in the oil.

### 3.5. Pigments (Chlorophylls and Carotenoids)

The highest chlorophyll degradation at T18 and after bottle opening was observed under the −18 °C storage condition for all three EVOOs (Figure 3, Table S4). Total chlorophyll decreased sharply immediately 72 h after treatment completion, with a lighter reduction only in HP oil, until its total degradation. A slight antioxidant activity of chlorophylls in the dark was reported, which was attributed to the possible donation of a hydrogen radical to break free-radical chain reactions [53]. Even if the impact of chlorophylls on the oxidative stability of VOO, i.e., in the presence of other antioxidants, seems to be rather limited, our results demonstrated that the degradation caused by the oxygen is highly comparable to that reported for more known antioxidants studied in the present work.

From 18 to 36 months of storage, total chlorophyll content remained almost the same at −18 °C in all three EVOOs, while at 4 °C, the trend was negative in HP and mostly in LP oils. The evolution of chlorophyll content under low temperature storage was not previously reported and the present study allowed to demonstrate how oil refrigeration, in the absence of oxygen, can restrain chlorophyll degradation.

After 18 months of storage, as well as after 36 months of storage, the β-carotene content remained almost unchanged in all EVOOs and storage conditions. Carotenoids (and especially β-carotene, as the most studied representative) have been generally recognized as inhibitors of photooxidation, thanks to their ability to quench singlet oxygens [7]. However, the antioxidative effect of carotenoids in EVOOs under conditions of autoxidation seems to be very limited or even negative, owing to their oxidation products, which may possibly react with the lipid substrate, and thus accelerate oxidation [54]. In the present study, β-carotene increase was correlated with the increase of oxidation after treatment completion, from 1 to 8 months, mainly in LP oil.

### 3.6. Fatty Acid Profiles

No significant changes in monounsaturated and polyunsaturated fatty acid percentages under different treatments and after 18 and 36 months storage were observed (Table S5). Oleic, palmitic, and linoleic acids remained in the normal range for the three EVOOs (~73, 12, and 6%, respectively), as well as the polyunsaturated linoleic and linolenic acids, that play a very important role on oil stability [54].

The present results are in agreement with studies reporting minor changes in fatty acids of oils stored at room temperature, under light, or at 2 °C in the dark [12,55,56], and no changes in polyunsaturated fatty acids for olive oils stored for 18 months in dark glass at room temperature [5]. No significant changes in both unsaturated and saturated fatty acids were reported either for low temperature storage conditions in the dark [20].

### 3.7. The Overall Analysis of EVOOs under Different Storage Conditions

**Figure 4:**
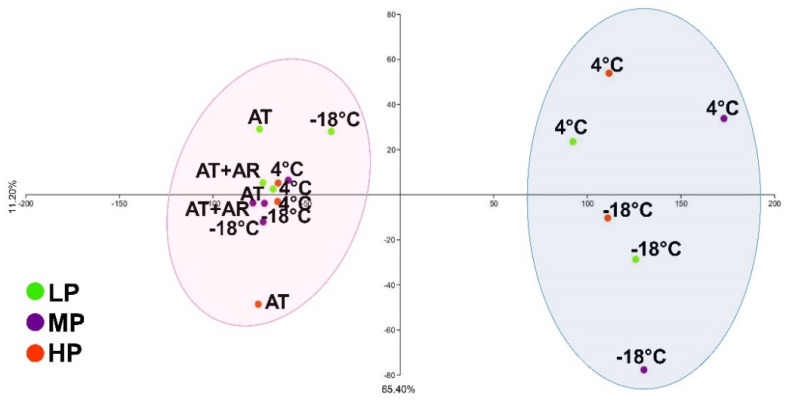
The scatter plot of the principal component analysis (PCA) using the percentage of reduction in content of twenty-two chemical parameters from T18 to T18-8 m (pink area) and between T18 and T36 (blue area) conditions. Colored circles indicate the type of oil, green: LP oil; purple: MP oil; orange: HP oil.

To explore the distribution pattern and visualize the effects of storage conditions on EVOOs, twenty-two variables, based on their percentage of reduction/increase during the time, were used to perform a PCA analysis (Figure 4). An evident and negative separation of samples according to their exposure to oxygen was observed. In PC1, explaining 65.40% of total variance, 14 out of 22 parameters were positive and, among them, the highest values were for 3,4-DHPEA-EA and p-HPEA-EA, squalene, β-sitosterol, β-sitostanol, α- and β-tocopherol, and total chlorophyll. PC2, with 11.20% of total variance, had 13 positive parameters, especially for oleacein, oleocanthal, 3,4-DHPEA-EA, acetoxypinoresinol, and campesterol. Eight months after treatment completion, PCA analysis grouped together all oil samples (with three out-grouping) in the negative plot of PC1 axis, independently from the storage condition, especially in MP oil. These results confirmed that autoxidation started after conservation and bottle opening, even for low temperature storage. After 18 months at AT, HP oil showed the highest degradation; noteworthy, in this oil, despite the highest concentration of antioxidants, after treatment completion and bottle opening, oxidation started very quickly. For what concerns the effect of argon on oil stability, it was observed that, after 18 months of conservation with and without modified atmosphere, only slight differences among oils were observed.

All three oils under the 4 °C storage condition were placed in the positive PCA plot area, showing high stability for all analyzed parameters, owing to a more suitable temperature and to oxygen limitation. These results are in accordance with [15], who reported that, by storing EVOOs at different temperatures (5, 15, 25, and 50 °C), their shelf-life was considerably extended at lower temperatures. Li et al. [7], evaluating the effects of different storage conditions (25, 4.5, and −27 °C) on EVOOs with different composition, showed that cold storage conditions (either 4.5 or −27 °C) were successful in retarding oxidation and hydrolysis level during storage, with no significant changes in the flavor over 4.5 months of storage. Mulinacci et al. [16] also reported that the phenolic fraction had lower oxidation after conservation at −23 °C, indicating a far superior quality of frozen EVOOs.

**Figure 5:**
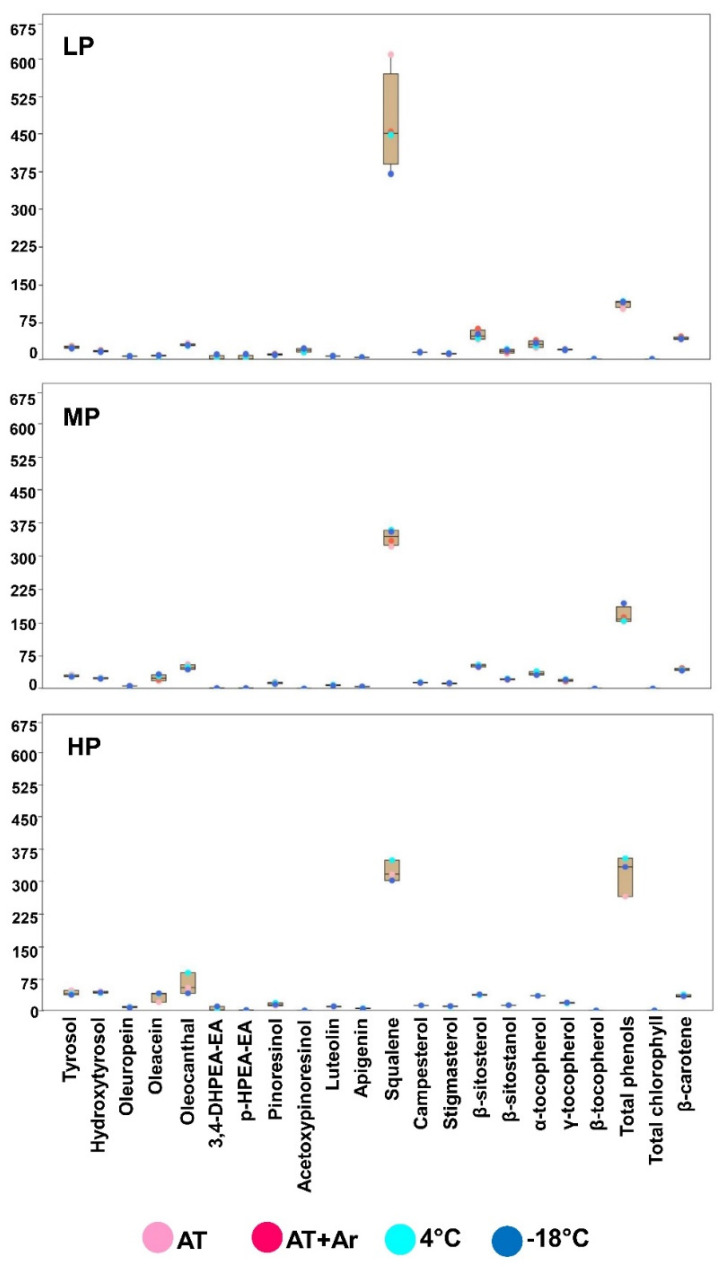
Box and jitter plot representing significant differences in the concentration of twenty-two chemical parameters at 8 months after bottles’ opening (T18-8 m) under different conditions. Colored circles indicate the different storage conditions; pink: AT; dark pink: AT + Ar; light blue: 4 °C; and dark blue: −18 °C.

The box plot analysis allowed identifying, among 22 variables, those that determined the response of oils to the storage treatments from T18 to T18-8 m (Figure 5). In general, chemical parameters more prone to change under different storage conditions were almost the same; however, the analyzed oils showed different responses at various temperatures. In LP oil, differences among storage conditions derived mainly from squalene, total phenols, and β-sitosterol. Squalene showed the highest amount at AT, followed by AT + Ar and 4 °C, while the lowest value was detected at −18 °C. In MP oil, the main variables among different storage conditions again were squalene and total phenols, where the highest amounts for both components were observed at −18 °C. Four chemical parameters played the main effect on HP oil stability under three storage conditions: squalene, total phenols, oleacein, and oleocanthal, and for all of them, the highest value of positive attributes was recorded for storage at 4 °C (Figure 5). The overall analysis on all oil samples and storage conditions showed 4 °C as the best temperature to preserve the oil for 36 months and, even after this long storage, all three oils were at the standard level for EVOO, as well as a good level for all 22 chemical components.

These results show that squalene, sterols, and tocopherols play a more important role as antioxidants in low phenolic EVOOs; instead, in high phenolic oil, phenols and especially secoiridoids are the main antioxidants.

Storage at −18 °C for 36 months showed that the three oils substantially maintained their characteristics, but, considering the energy and structural costs to maintain the cold chain, it can be assumed that 4 °C should be considered as the best condition for EVOO long storage.

### 3.8. Organoleptic Evaluation

**Figure 6:**
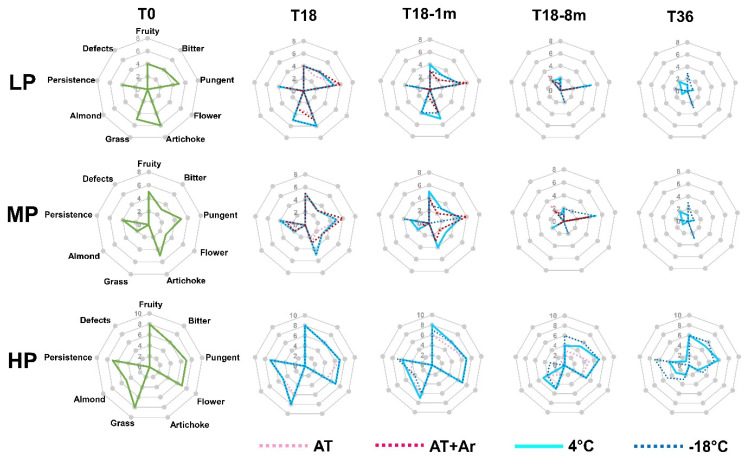
Radar chart reporting the median of fifteen organoleptic evaluations (three replicas X oil X panel members) carried out on EVOOs at different time points (T0, T18, T18-1 m, T18-8 m, and T36).

An organoleptic evaluation was carried out at every time point (T0, T18, T18-72 h, T18-1 m, T18-8 m, T36) (Figure 6) (owing to the lack of differences between T18 and T18-72 h, this latter time point was not reported in the figure).

Before storage, LP and MP oils showed good levels of fruitiness, bitterness, and pungency, but a general low level of flavors, while HP oil showed very good taste and flavor. After 18 months of conservation at 4 °C and −18 °C, flavors remained almost unchanged, while a reduction in flavor and start of grassy occurred at AT. The same was observed at 1 month after storage completion, when oils stored at 4 °C and at −18 °C again did not have considerable changes, while oils stored at AT degraded significantly. The decrease in fruitiness, grassy, and almond at T18-8 m was perceived both under 4 °C and −18 °C storage conditions. In fact, at T18-8 m, defects were perceived at AT, AT + Ar, and 4 °C for LP and MP oils, while at −18 °C, even if bitterness and flavors almost disappeared, no defects were perceived in all analyzed EVOOs. It was interesting to note that, in HP oil, even 8 months after LTS, no defects were noticed and this oil still had pungency, fruitiness, flavor, and bitterness, especially for that treated at −18 °C.

The oil samples at −18 °C did not have any defect even after 36 months of conservation; moreover, in HP oil, artichoke flavor was detected, with a good perception of fruitiness, pungency, and persistence.

In the present study, only HP oil eight months after 18 months of storage completion and even after 36 months of storage under a low temperature preserved the sensorial attributes. The impact of storage conditions on the sensory attributes seems to be strictly connected to the initial content of hydrophilic phenols in the EVOO and only HP oil may avoid defects and maintain gustatory and olfactory sensations.

Among low temperature treatments, −18 °C was a bit more effective than 4 °C in preserving the persistency, fruitiness, bitterness, and some olfactory sensations.

The hydrophilic phenols greatly influence the taste quality (bitter and pungency attributes), as well as the beneficial biological activity and oxidative stability of EVOOs [57,58]. Among these phenols, oleacin and oleuropein aglycone are mainly responsible for the bitter taste, whereas oleocanthal is the main agent responsible for pungency. In fact, the significant reduction in oleacin and oleuropein affected the bitterness during conservation, especially in LP and MP oils, while oleocanthal had the minimum reduction among secoiridoids, allowing to perceive the pungency even 8 months after storage end, especially in HP oil. Some flavor attributes (i.e., fresh fruity and artichoke) may be related to the decrease of some secoiridoids during storage [59].

## 4. Conclusions

Long (18 months) and very-long (36 months) storage under different conditions (AT, AT + Ar, 4 °C, and −18 °C) was evaluated for low, medium, and high-phenolic EVOOs, in order to verify their effects on oil preservation at different time points (0 and 72 h, 1 and 8 months) after treatment completion.

The decay along all storage treatments was mostly evident for phenolic compounds, while tocopherols, squalene, and sterols have undergone a low reduction and fatty acids showed negligible alteration. The use of argon in the bottle headspace at an ambient temperature could be recommended only for 18 months of storage, whereas, later on, the use of modified atmosphere did not allow significantly reducing EVOO degradation. On the contrary, cold storage was successful in retarding the oxidation and hydrolysis at least until the oxygen availability was limited. After 18 months of storage and bottle opening, in fact, degradation rates increased in all treatments and most metabolites declined, independently from the previous storage treatment. The 36 months of storage definitively showed the effect of low temperatures to avoid oxidation and preserve the metabolite profile of EVOOs, while the organoleptic profile was preserved only for oils with a high initial content of hydrophilic phenols. The conservation of EVOOs at −18 °C could be applied for high-quality oils (with a high level of phenols and a good organoleptic profile) to avoid their degradation during storage and delivery chain.

To maintain the nutraceutical properties of EVOOs, mild cold at 4 °C was the best storage temperature, especially during the first 18 months of conservation, in comparison with the conventional storage or even at −18 °C, also considering the elevated costs and energy use for maintaining freezing temperatures during long-term storage and delivery.

These results demonstrated that it is possible to preserve EVOO quality for a very long time, up to 3 years, if proper storage technologies are applied. The present study has provided important knowledge on the change in key chemical and organoleptic parameters in extra virgin olive oils during the storage. A new strategy to better preserve the characteristics of EVOOs at industrial and commercial level has been provided.

## Data Availability

The data presented in this study are available here and in the Supplementary Material.

## Supplementary Materials

The following are available online at https://www.mdpi.com/article/10.3390/foods10081945/s1: Figure S1: Schematic view of the experimental design. At each time point, three bottles were used for the analyses, Figure S2: The scatter plot (A) and biplot (B) of the principal component analysis (PCA), using the percentage of reduction in content of 11 phenolic compounds in EVOOs from T18 to T18-8 m (pink area) and between T18 and T36 (blue area) conditions. Colored circles indicate the type of oil, green: LP oil; purple: MP oil; orange: HP oil, Table S1: Free acidity (% of oleic acid), peroxide value (meq O_2_ kg^−1^), and total phenols (mg kg^−1^ of oil) of three kinds of oil in different storage conditions at each time point. Different letters (*p* < 0.01 and *p* < 0.05) show significant differences in each single column, Table S2: K_232_ and K_270_ extinction coefficients of three kinds of oils in different storage conditions at each time point. Different letters (*p* < 0.01 and *p* < 0.05) show significant differences in each single column, Table S3: The *p*-value of ANOVAs among different time points for three kinds of oil. The non-significant and *p* < 0.05 were shown by bold superscript ns and *, respectively, and all other values were significant at *p* < 0.01, Table S4: The *p*-value of ANOVAs among different time points for three kinds of oil. The non-significant and *p* < 0.05 were shown by bold superscript ns and *, respectively, and all other values were significant at *p* < 0.01, Table S5: Mean value of the main fatty acids in three different oil samples, under four storage conditions and in six time points.
