# Supplementary material for: Evolution of Extra Virgin Olive Oil Quality under Different Storage Conditions
Source: Foods. 2021 Aug 21;10(8):1945. doi: 10.3390/foods10081945 (PMC8391879; doi:10.3390/foods10081945)
Supplement: Supplementary file 1 [file foods-10-01945-s001.zip › foods-1313906-supplementary.pdf]

Supplementary Material

Evolution of extra virgin olive oil quality under different storage conditions

Soraya Mousavi[1], Roberto Mariotti[1*], Vitale Stanzione[2], Saverio Pandolfi[1], Valerio Mastio[3], Luciana Baldoni[1], Nicolò G. M. Cultrera[1]

[1] Institute of Biosciences and Bioresources, National Research Council, 06128 Perugia, Italy; soraya.mousavi@ibbr.cnr.it (S.M.); roberto.mariotti@ibbr.cnr.it (R.M.); saverio.pandolfi@ibbr.cnr.it (S.P.); luciana.baldoni@ibbr.cnr.it (L.B.); : niccolo.cultrera@ibbr.cnr.it (N.C.)

[2] Institute for Agricultural and Forest Systems of the Mediterranean, National Research Council, 06128 Perugia, Italy; vitale.stanzione@cnr.it (V.S.)

[3] Estación Experimental Agropecuaria San Juan, Instituto Nacional de Tecnología Agropecuaria (INTA), Consejo Nacional de Investigaciones Científicas y Técnicas (CONICET), San Juan, Argentina; mastio.valerio@inta.gob.ar (V.M.)

* Correspondence: roberto.mariotti@ibbr.cnr.it; Tel.: +39 075 5014809

Table S1. Free acidity (% of oleic acid), peroxide value (meq O2 kg^{-1}) and total phenols (mg kg^{-1} of oil) of three kinds of oil in different storage conditions in each time point. Different letters (P < 0.01 and P < 0.05) show significant differences in each single column.

**LP**	**T0**	**T18***	**T18-72h**	**T18-1m**	**T18-8m**	**T36***
			Free acidity			
**AT**	0.17	0.21	0.26a	0.29a	0.35	0.42a
**AT+Ar**	0.15	0.19	0.22ab	0.25ab	0.33	0.41a
**4°C**	0.16	0.18	0.24a	0.26ab	0.35	0.19b
**-18°C**	0.16	0.18	0.20b	0.23b	0.31	0.18b
			Peroxide value			
**AT**	5.43	14.49a	16.37a	16.63a	31.55a	32.85a
**AT+Ar**	5.20	6.37c	6.97c	8.54b	24.60b	28.35b
**4°C**	5.54	8.68b	12.64b	17.15a	35.19a	17.60c
**-18°C**	5.86	7.22bc	11.28b	15.76a	33.50a	12.24d
			Total phenols			
**AT**	104.887	143ab	125.97b	105.07	102.19	54.62c
**AT+Ar**	108.60	154.48a	125.00b	115.90	115.17	64.24c
**4°C**	109.50	162.16a	148.59a	122.87	117.74	103.95a
**-18°C**	107.67	124.46b	129.04ab	120.74	115.97	85.03b
**MP**	**T0**	**T18***	**T18-72h**	**T18-1m**	**T18-8m**	**T36***
			Free acidity			
**AT**	0.21	0.26a	0.27	0.29a	0.35	0.45a
**AT+Ar**	0.21	0.23b	0.26	0.27ab	0.36	0.40a
**4°C**	0.20	0.22b	0.23	0.24b	0.33	0.18b
**-18°C**	0.19	0.23b	0.25	0.24b	0.34	0.19b
			Peroxide value			
**AT**	4.40	10.74ab	11.17b	12.29bc	31.86	38.62a
**AT+Ar**	4.44	13.16a	14.01a	15.02ab	29.97	35.25b
**4°C**	4.77	9.58b	11.59ab	15.50a	31.14	15.60c
**-18°C**	4.52	8.19b	8.92b	11.47c	26.38	9.60d
			Total phenols			
**AT**	206.17	235.86	249.61	194.32	153.10b	100.26b
**AT+Ar**	206.37	270.05	235.39	193.15	163.69b	112.21b
**4°C**	206.17	233.97	237.31	203.62	154.94b	178.30a
**-18°C**	206.17	252.87	263.14	209.62	195.41a	133.8ab
**HP**	**T0**	**T18***	**T18-72h**	**T18-1m**	**T18-8m**	**T36***
			Free acidity			
**AT**	0.25a	0.26a	0.27	0.27	0.35	0.39a
**4°C**	0.25a	0.26a	0.25	0.26	0.34	0.25b
**-18°C**	0.18b	0.20b	0.22	0.26	0.34	0.23b
			Peroxide value			
**AT**	2.62	11.16a	14.11a	18.16a	29.22a	35.22a
**4°C**	2.78	4.97b	6.21b	9.22b	24.54b	11.60b
**-18°C**	2.62	3.88b	5.73b	9.07b	21.88b	4.72c
			Total phenols			
**AT**	469.77	441.51	439.21b	413.79	269.16b	140.55b
**4°C**	477.22	496.84	507.26ab	452.93	359.68a	421.39a
**-18°C**	504.56	544.07	568.98a	506.44	339.31a	382.17a

*Closed bottles.

Maximum values of EVOO quality parameters (EU Reg. 2015/1830): Free acidity, 0.30%; Peroxide value, 20 meq O2 kg^{-1}.

Table S2. K_{232} and K_{270} extinction coefficients of three kinds of oils in different storage conditions in each time point. Different letters ($P < 0.01$ and $P < 0.05$) show significant differences in each single column.

**LP**	**T0**	**T18***	**T18-72h**	**T18-1m**	**T18-8m**	**T36***
			K_{232}			
**AT**	0.071	0.09	0.08a	0.08a	9.71	11.4b
**AT+Ar**	0.071	0.10	0.04c	0.05c	10.32	12.25a
**4°C**	0.071	0.11	0.06b	0.06b	10.73	0.11c
**-18°C**	0.071	0.10	0.04c	0.04c	9.76	0.01c
			K_{270}			
**AT**	0.06	0.08a	0.09bc	0.08	2.67a	2.9a
**AT+Ar**	0.06	0.07ab	0.14a	0.08	1.98b	2.69a
**4°C**	0.06	0.06c	0.07c	0.09	2.95a	0.07b
**-18°C**	0.06	0.06bc	0.12ab	0.10	2.59a	0.02b
**MP**	**T0**	**T18***	**T18-72h**	**T18-1m**	**T18-8m**	**T36***
			K_{232}			
**AT**	0.05	0.1	0.05	0.03a	9.95	11.21b
**AT+Ar**	0.05	0.11	0.05	0.02b	9.55	11.39a
**4°C**	0.05	0.11	0.05	0.03a	9.89	0.12c
**-18°C**	0.05	0.10	0.05	0.03ab	9.88	0.05d
			K_{270}			
**AT**	0.05	0.08	0.09	0.17a	2.00b	2.92b
**AT+Ar**	0.05	0.08	0.09	0.16a	2.56a	3.12a
**4°C**	0.05	0.08	0.09	0.13b	2.54a	0.08c
**-18°C**	0.05	0.07	0.08	0.13b	2.35ab	0.08c
**HP**	**T0**	**T18***	**T18-72h**	**T18-1m**	**T18-8m**	**T36***
			K_{232}			
**AT**	0.04	0.10	0.05	0.04	9.52	11.48a
**4°C**	0.04	0.08	0.04	0.03	10.64	0.08b
**-18°C**	0.04	0.08	0.04	0.03	9.34	0.01b
			K_{270}			
**AT**	0.04	0.11a	0.21a	0.08	2.46a	2.88a
**4°C**	0.04	0.08b	0.16b	0.08	2.26a	0.08b
**-18°C**	0.04	0.08b	0.15b	0.08	1.82b	0.01c

*Closed bottles.

Maximum values of EVOO quality parameters (EU Reg. 2015/1830): K_{270}, 0.22; K_{232}, 2.50.

Table S3. The p-value of ANOVA analyses among different time points for three kinds of oil. The non-significant and $p < 0.05$ were showed by bold superscript ns and *, respectively, all other values were significant at $p < 0.01$.

**Compound**	**AT**	**AT + Ar**	**4°C**	**-18°C**
Tyrosol-LP	7.12E-07	3.54E-06	7.82E-06	7.61E-07
Tyrosol-MP	3.31E-05	2.45E-06	7.97E-06	2.32E-06
Tyrosol-HP	3.98E-06		3.36E-06	2.51E-06
Hydroxytyrosol-LP	5.07E-05	1.84E-05	1.38E-05	1.39E-05
Hydroxytyrosol-MP	0.000228	0.000107	2.57E-06	1.1E-05
Hydroxytyrosol-HP	1.33E-05		9.57E-06	2.04E-05
Oleuropein-LP	4.98E-05	1.24E-06	4.27E-05	8.79E-07
Oleuropein-MP	2.84E-05	9.5E-06	1.04E-05	1.15E-05
Oleuropein-HP	3.08E-06		4.72E-06	9.36E-07
Oleacein-LP	0.001473	0.000193	1.25E-05	0.000112
Oleacein-MP	1.37E-05	8.74E-06	0.000389	$0.142425^{\mathbf{ns}}$
Oleacein-HP	7.99E-06		0.003418	0.000408
Oleocanthal-LP	$0.085834^{\mathbf{ns}}$	0.044468^{*}	0.000645	0.002858
Oleocanthal-MP	$0.091088^{\mathbf{ns}}$	0.012771^{*}	0.01089^{*}	0.000585
Oleocanthal-HP	8.81E-06		0.004224	0.00018
3,4-DHPEA-EA-LP	8.61E-05	2.98E-05	1.03E-05	9.8E-06
3,4-DHPEA-EA-MP	0.000153	5.36E-06	1.84E-05	7.44E-06
3,4-DHPEA-EA-HP	7.11E-06		1.59E-05	1.97E-05
p-HPEA-EA-LP	0.000103	0.00015	6.63E-06	0.000453
p-HPEA-EA-MP	4.04E-05	6.35E-05	9.31E-06	1.96E-05
p-HPEA-EA-HP	0.000172		6.08E-06	4.47E-05
Pinoresinol-LP	3.25E-05	6.34E-05	6.51E-05	1.88E-05
Pinoresinol-MP	7.28E-05	4.73E-05	1.8E-05	0.000379
Pinoresinol-HP	0.000326		1.9E-05	7.4E-06
Acetoxypinoresinol-LP	0.027117^{*}	6.02E-05	0.000583	0.007268
Acetoxypinoresinol-MP	1.46E-05	1.37E-05	5.23E-06	1.26E-05
Acetoxypinoresinol-HP	6.54E-06		9.63E-05	2.65E-05
Luteolin-LP	0.001544	0.001613	0.009755	0.01175^{*}
Luteolin-MP	$0.102757^{\mathbf{ns}}$	0.024285^{*}	0.039881^{*}	0.003388
Luteolin-HP	0.000667		0.000226	0.004262
Apigenin-LP	0.002763	0.001265	0.001563	0.001679
Apigenin-MP	0.00523	0.00843	0.019822^{*}	0.00036
Apigenin-HP	3.68E-05		$0.061126^{\mathbf{ns}}$	0.002052
α-Tocopherol-LP	0.000101	2.7E-05	1.2E-05	2.79E-05
α-Tocopherol-MP	0.000232	9.95E-05	1.72E-05	1.81E-05
α-Tocopherol-HP	5.9E-06		0.00016	7.68E-06
β-Tocopherol-LP	1.46E-05	1.36E-05	1.34E-05	1.17E-05
β-Tocopherol-MP	4.51E-06	6.51E-06	8.75E-06	6.23E-05
β-Tocopherol-HP	0.000152		7.43E-05	8.11E-05
γ-Tocopherol-LP	0.001166	0.000242	0.000403	0.000496
γ-Tocopherol-MP	0.001374	0.001025	0.0014	0.00328
γ-Tocopherol-HP	0.000476		0.000399	0.000351

Table S4. The p-value of ANOVA analyses among different time points for three kinds of oil. The non-significant and $p < 0.05$ were showed by bold superscript ns and *, respectively, all other values were significant at $p < 0.01$.

Compound	AT	AT + Ar	4°C	-18°C
Squalene-LP	2.2E-05	2.45E-06	2.11E-05	1.98E-05
Squalene-MP	1.36E-05	1.25E-06	7.55E-05	4.6E-05
Squalene-HP	1.24E-05		3.38E-05	9.19E-06
β-Sitosterol-LP	1.12E-05	0.000209	2.95E-05	2.64E-05
β-Sitosterol-MP	3.67E-05	2.96E-05	2.98E-05	1.96E-05
β-Sitosterol-HP	3.51E-06		9.4E-06	2.25E-05
β-Sitostanol-LP	2.21E-05	5.47E-05	0.003314	0.002316
β-Sitostanol-MP	0.001808	0.000503	0.000629	0.000213
β-Sitostanol-HP	0.000298		0.001492	2.51E-05
Campesterol-LP	0.009968	0.000792	0.003523	0.001925
Campesterol-MP	0.014246*	0.000857	0.073725$^{\mathbf{ns}}$	0.00594
Campesterol-HP	0.000534		0.008855	0.00044
Stigmasterol-LP	0.030178*	0.02969*	0.023058*	0.071656$^{\mathbf{ns}}$
Stigmasterol-MP	0.000879	0.000724	0.039262*	0.024456*
Stigmasterol-HP	0.000679		0.000975	0.052048$^{\mathbf{ns}}$
Total Chlorophyll-LP	1E-06	5.93E-06	5.12E-06	1.24E-05
Total Chlorophyll-MP	9.56E-06	2.29E-05	2.41E-06	1.73E-06
Total Chlorophyll-HP	6.39E-05		3.56E-06	1.36E-05
β-Carotene-LP	2.97E-05	8.31E-05	2.57E-05	0.000418
β-Carotene-MP	4.9E-05	5.63E-05	5.99E-05	2.48E-05
β-Carotene-HP	0.00049		4.91E-05	0.000137

Table S5: Mean value of the main fatty acids in three different oil samples, under four storage conditions and in six time points.

**LP-Storage condition**	**Time point**	**C16:0**	**C18:1**	**C18:2**	**C18:3**
AT	T0	13.48	78.37	7.78	1.03
AT	T18	13.34	78.00	7.83	0.76
AT	T18-72h	12.93	76.39	7.63	0.73
AT	T18-1m	13.76	77.88	7.73	0.69
AT	T18-8m	14.00	76.90	7.53	0.70
AT + Ar	T0	13.22	79.87	7.51	0.72
AT + Ar	T18	13.29	77.11	7.83	0.79
AT + Ar	T18-72h	13.65	77.72	7.81	0.69
AT + Ar	T18-1m	13.99	77.83	7.87	0.71
AT + Ar	T18-8m	13.10	78.27	7.60	0.69
4°C	T0	13.76	78.21	7.59	0.96
4°C	T18	13.79	77.62	7.63	0.72
4°C	T18-72h	13.54	77.65	7.68	0.69
4°C	T18-1m	14.98	76.70	7.29	0.72
4°C	T18-8m	15.08	76.26	7.20	0.70
4°C	T36	15.99	76.49	7.22	0.75
-18°C	T0	13.76	78.21	7.59	1.01
-18°C	T18	13.74	77.72	7.67	0.77
-18°C	T18-72h	13.02	77.30	7.53	0.71
-18°C	T18-1m	13.64	77.83	7.84	0.75
-18°C	T18-8m	15.15	76.19	7.75	0.72
-18°C	T36	15.11	76.62	7.40	0.73
MP-Storage condition	Time point	**C16:0**	**C18:1**	**C18:2**	**C18:3**
AT	T0	15.01	76.00	7.92	0.85
AT	T18	15.05	76.80	7.17	0.70
AT	T18-72h	15.63	76.14	7.61	0.69
AT	T18-1m	16.01	76.17	7.13	0.65
AT	T18-8m	15.17	76.17	7.96	0.66
AT + Ar	T0	15.21	76.13	7.54	0.98
AT + Ar	T18	15.68	76.30	7.14	0.72
AT + Ar	T18-72h	15.80	76.54	6.99	0.64
AT + Ar	T18-1m	15.11	76.80	7.20	0.66
AT + Ar	T18-8m	15.80	76.21	7.24	0.68
4°C	T0	15.01	76.42	7.23	0.85
4°C	T18	15.36	76.61	7.37	0.69
4°C	T18-72h	15.88	76.22	7.22	0.64
4°C	T18-1m	15.86	76.29	7.26	0.66
4°C	T18-8m	15.83	76.35	7.14	0.63
4°C	T36	15.85	76.27	7.16	0.68
-18°C	T0	16.01	76.21	7.03	0.85
-18°C	T18	15.77	76.01	7.35	0.68
-18°C	T18-72h	15.94	76.04	7.33	0.70
-18°C	T18-1m	15.13	77.01	7.26	0.67
-18°C	T18-8m	15.60	76.42	7.15	0.69

-18°C	T36	15.76	76.42	7.19	0.74

HP-Storage condition	Time point	**C16:0**	**C18:1**	**C18:2**	**C18:3**
AT	T0	13.85	78.97	6.35	0.84
AT	T18	12.65	79.88	6.49	0.66
AT	T18-72h	13.08	78.84	6.87	0.65
AT	T18-1m	13.46	78.94	6.43	0.61
AT	T18-8m	13.51	78.73	6.25	0.59
4°C	T0	13.55	78.39	6.74	0.89
4°C	T18	13.88	78.95	6.49	0.65
4°C	T18-72h	13.81	79.30	6.31	0.63
4°C	T18-1m	14.25	78.20	6.55	0.65
4°C	T18-8m	13.70	78.02	7.21	0.64
4°C	T36	13.96	78.24	6.70	0.70
-18°C	T0	13.85	78.25	6.55	0.89
-18°C	T18	13.91	78.41	6.69	0.69
-18°C	T18-72h	13.84	78.42	6.63	0.69
-18°C	T18-1m	14.48	78.23	6.58	0.69
-18°C	T18-8m	13.71	79.10	6.45	0.66
-18°C	T36	14.15	78.33	6.48	0.70

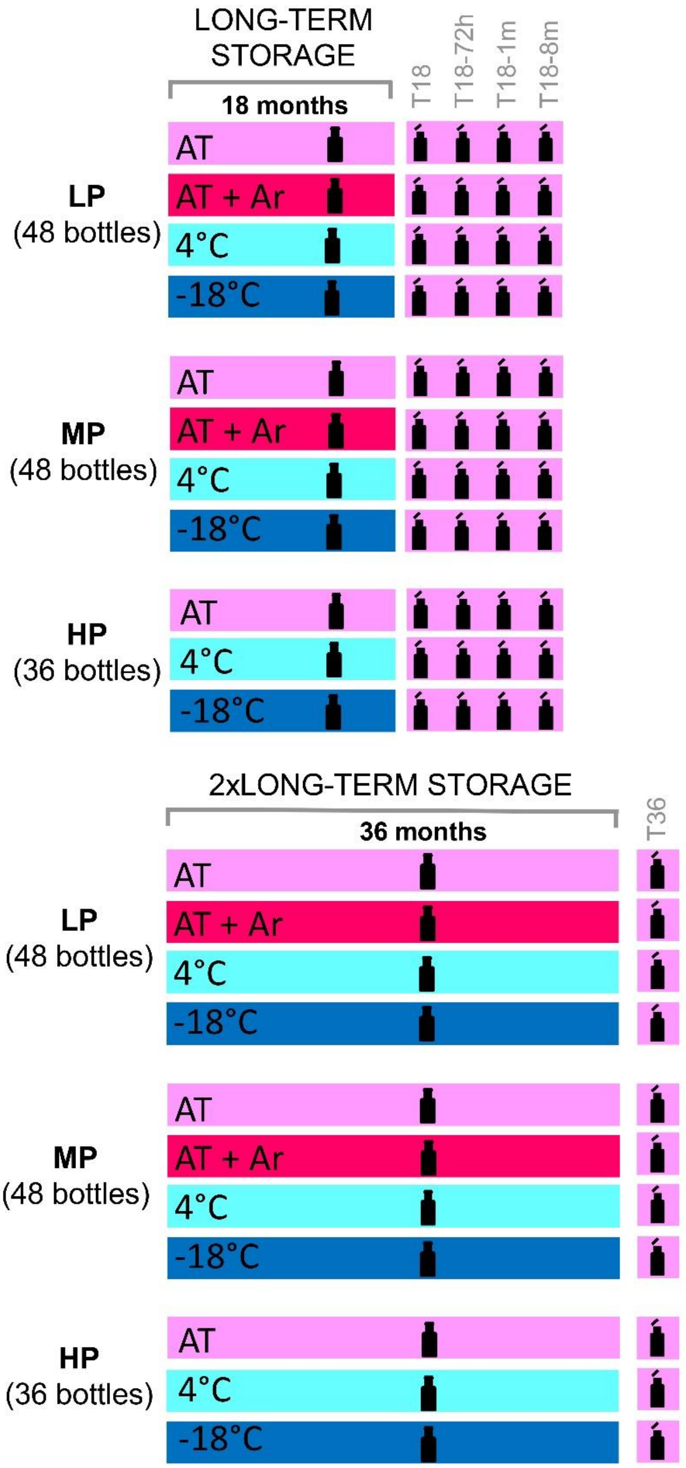

Figure S1: Schematic view of the experimental design. At each time point, three bottles were used for the analyses.

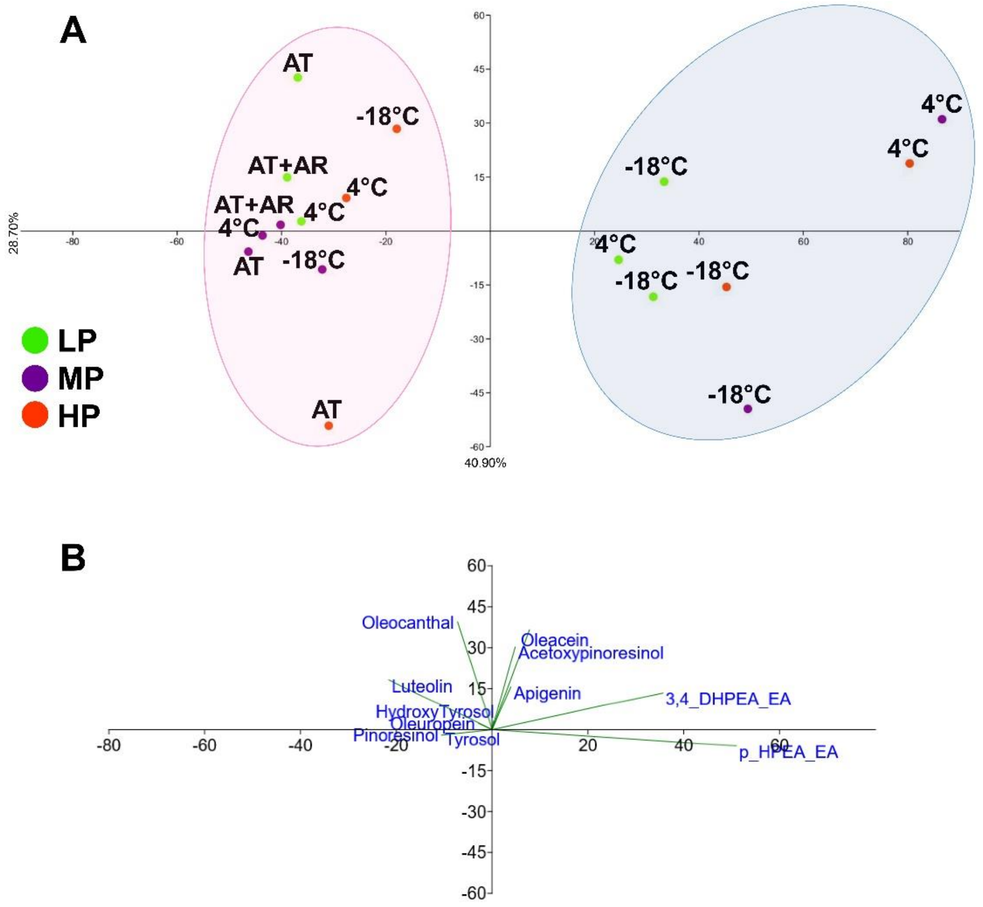

Figure S2: The scatter plot (A) and biplot (B) of the principal component analysis (PCA), by using the percentage of reduction in content of 11 phenolic compounds in EVOOs from T18 to T18-8m (pink area) and among T18 and T36 (blue area) conditions. Colored circles indicate the type of oil, green: LP oil; purple: MP oil; orange: HP oil.
